# Evaluation of the Abbott Alinity m STI assay for diagnosis of the primary causes of sexually transmitted infections in the United States

**DOI:** 10.1016/j.plabm.2023.e00332

**Published:** 2023-08-22

**Authors:** Colette Matysiak, Annie Cheng, James E. Kirby

**Affiliations:** a Harvard Medical School, Boston, MA, USA; b Beth Israel Deaconess Medical Center, USA

**Keywords:** *Chlamydia trachomatis*, *Neisseria gonorrhoeae*, *Trichomonas vaginalis*, *Mycoplasma genitalium*, Diagnostics, Syndromic panel

## Abstract

**Objectives:**

The sexually transmitted infections, *Chlamydia trachomatis* (CT), *Neisseria gonorrhoeae* (NG), *Trichomonas vaginalis* (TV), and *Mycoplasma genitalium* (MG), have similar risk factors and symptoms, supporting use of a quadruplex test as an efficient diagnostic modality.

We assessed the clinical and analytical performance of the Abbott Alinity m STI assay to detect these pathogens.

**Design and methods:**

Urine and genital swabs from 142 patient samples were tested from an adult outpatient population in the Northeast United States. The positive and negative percent agreement for CT, NG, and TV were determined by comparison with the Hologic Panther Aptima assay. The analytical sensitivity was determined through serial dilution of standards for CT, NG, TV, and MG in negative urine and swab matrix.

**Results:**

The positive and negative percent agreement of the Alinity m assay in comparison with the Hologic Panther Aptima assay were, respectively: CT [100.0% (90.6–100.0%) and 99.1% (94.8–100.0%)], NG [100.0% (89.6–100.0%) and 99.1% (94.9–100.0%)]; and TV [96.3% (81.7–99.8%) and 99.1% (95.2–100.0%)]. The limits of detection in urine and swab matrix were, respectively: CT ≤ 5, ≤1; NG ≤ 5, ≤5; TV ≤ 0.5, ≤0.5; and MG ≤ 500, ≤250 genome copies/mL.

**Conclusions:**

The Alinity m assay demonstrated excellent performance characteristics and identifies CT, NG, and TV accurately compared with a well-established comparator.

## Highlights

• The Abbott Alinity m STI assay was evaluated with clinical urogenital samples.
• The test detects four major bacterial causes of STI's at the same time.
• The Alinity had high percent agreement with the Hologic Panther Aptima assay.
• The Alinity assay was able to detect low levels of each STI.
• The assay detected up to three coinfections in individual clinical samples.

## 1 Introduction

Approximately 20% of the U.S. population has a sexually transmitted infection (STI), and 26 million new infections are acquired each year [1]. In the U.S., *Trichomonas vaginalis* (TV), *Chlamydia trachomatis* (CT), and *Neisseria gonorrhoeae* (NG) are the three most common curable STIs. Only human papillomavirus (HPV) infection has a greater annual incidence (13 million) than TV (6.9 million), CT (4 million), or NG (1.6 million) [1,2]. CT, NG, and TV can present with similar signs and symptoms including itching, burning, discharge, and bleeding. However, more than half of infections are asymptomatic [[3], [4], [5]]. If left untreated, initially asymptomatic infections have been associated with pelvic inflammatory disease, ectopic pregnancy, infertility, premature birth, and reactive or infectious arthritis [6,7]. There is growing evidence that *Mycoplasma genitalium* (MG) contributes to 20–25% of non-NG non-CT urethritis; 40% of persistent or recurrent urethritis; and is detected in 4–22% of women with pelvic inflammatory disease [8,9]. Additional research is needed to clarify the association between MG and these diseases.

Given the considerable disease burden and the overlapping symptoms and risk factors for CT, NG, TV, and MG, a high throughput quadruplex assay would enable efficient diagnosis and surveillance. We therefore set out to determine the clinical and analytical performance of the recently FDA-cleared Alinity m STI assay by evaluating the positive and negative percent agreement for CT, NG, and TV compared with testing results from Hologic Panther Aptima assays. We also characterized coinfection with MG and determined the Alinity m limit of detection in genital swabs and urine matrix for all four pathogens.

## 2 Methods

### 2.1 Study design and population

This study was conducted to evaluate the recently FDA-cleared Alinity m STI Assay in an adult outpatient population in a metropolitan area in the Northeast United States. Samples were collected as a part of routine patient care from females and males as urine or genital swabs (cervical, vaginal, and urethral) in Aptima transport media. Samples were tested initially on the Hologic Panther Aptima assay and reanalyzed on the Alinity m as part of an evaluation for potential adoption of the new technology in our clinical laboratory. The study was determined to be exempt research by the Beth Israel Deaconess Medical Center Committee on Clinical Investigation.

### 2.2 Diagnostic tests and Procedures

The Alinity m STI assay is a nucleic acid amplification test (NAAT) that employs nucleic acid capture, PCR amplification, and real-time fluorescent detection. It reports qualitative detection of CT, TV, and MG ribosomal RNA, and NG DNA targets results, based on cycle threshold and maxRatio cutoffs [10] for both analytes and internal and cellular controls. The Alinity m STI assay is run on the Alinity m System, which can concurrently run multiple other Alinity m assays [11]. To evaluate the Alinity m positive and negative percent agreement for CT, NG, and TV, we tested a combination of samples, positive and negative for these analytes as determined by the Hologic Panther Aptima assay. The Aptima assay is a NAAT that detects ribosomal RNA for CT and NG in combination or ribosomal RNA for TV using target capture, transcription-mediated amplification, and chemiluminescent DNA probe detection reported as relative light units [12,13].

Aptima positive samples were collected between October 2020 and April 2021 and stored frozen at −80 °C for two to eight months until retesting on the Alinity m system. Aptima negative samples were collected in June 2021 and stored at 4 °C for up to 7 days until retesting on the Alinity m system. Samples that tested positive for CT, NG, or TV by Aptima and then negative by Alinity m were retested on both systems. Three samples that initially tested positive by Aptima (2 NG, and 1 TV) but tested negative upon repeat testing by Aptima, were assumed to have degraded during storage, and were removed from the analysis.

Currently our lab does not perform clinical MG testing. Therefore, the rate of detection of MG in samples testing positive or negative for CT, NG, and/or TV was determined on the Alinity m without an Aptima comparator result.

### 2.3 Limit of detection

The limit of detection (LoD) was evaluated for CT, NG, TV, and MG using analytical standards from Microbiologics (St. Cloud, MN; inactivated CT, NG, and TV, catalog #8228, and MG, catalog #8240). The inactivated pellets from Microbiologics were first reconstituted in 1 mL of Abbott multi-Collect media providing a stock concentration of 2000 for CT; 20,000 for NG; 2,000 for TV; and 10,000 for MG genome copies/mL. The stock samples were either serially diluted in Abbott multi-Collect media and then mixed 50:50 with pooled urine that had previously tested negative for all four pathogens, or serially diluted in patient negative swab matrix diluted 1:10 in Abbott multi-Collect media. An initial screen was performed with four replicates at doubling dilutions to determine the approximate LoD. The ranges tested were the same for urine and swab matrix unless noted: CT 0.1 to 100; NG 1 to 1000; TV 0.1 to 100; MG 10 to 1000 in urine and 50 to 1000 in swab matrix genome copies/mL. We then performed a secondary screen using a narrower concentration range with twenty replicates per dilution. The LoD was defined as the lowest concentration tested for which the target was detected in ≥95% of replicates. The ranges tested were the same for urine and swab matrix unless noted: CT 0.5 to 5; NG 0.5 to 50 for urine and 1 to 10 for swab matrix; TV 0.05 to 5; MG 250 to 500 genome copies/mL.

### 2.4 Statistical methods

Positive and negative percent agreement were calculated with GraphPad Prism version 9 using Wilson-Brown 95% confidence intervals.

## 3 Results

A total of 142 samples from different patients were collected in Aptima transport medium and analyzed in this study including genital swabs and urine samples as delineated in Table 1. The positive and negative percent agreement for the Alinity m STI assay compared with the Hologic Panther Aptima assay were, respectively 100.0% (95% Confidence Interval [CI]: 90.6%–100.0%) and 99.1% (95% CI: 94.8%–100.0%) for CT; 100.0% (95% CI: 89.6%–100.0%) and 99.1% (95% CI: 94.9%–100.0%)] for NG; and 96.3% (95% CI: 81.7%–99.8%) and 99.1% (95% CI: 95.2%–100.0%) for TV (see Table 2). In four samples, when there was a discrepancy between the Alinity m and Aptima results, the samples showed either very low relative light units (RLU, Aptima) and/or high cycle number values (CN, Alinity m), near the respective assay cutoffs for positivity. The discrepant CT sample was NG + on both Aptima and Alinity, but CT + only on Alinity with an RLU of 1000 on Aptima (called for NG + signal only) and with Alinity CN values of 38.69 and 32.68 for NG and CT, respectively. The discrepant NG sample (Aptima negative, Alinity positive) had an RLU of 10 and CN of 37.81. The first discrepant TV sample (Aptima negative, Alinity positive) had an RLU of 1 and CN of 34.44. The second discrepant TV sample (Aptima positive, Alinity negative) had an RLU of 145 and CN of 35.44.

**Table 1:** Distribution of samples by patient gender and sample type (genital swab, urine), with *Chlamydia trachomatis*, *Neisseria gonorrhoeae*, and *Trichomonas vaginalis* status as determined by the Hologic Panther Aptima assay.

Sample Type	*C. trachomatis* Positive	*N. gonorrhoeae* Positive	*T. vaginalis* Positive	Negative	Total Samples Evaluated^a^
Female Cervical Swab	4	4	6	15	28
Female Vaginal Swab	10	5	8	22	43
Female NS Swab	1	2	1	2	4
Male Urethra Swab	2	4	0	0	6
**Total Swab**	**17**	**15**	**15**	**39**	**81**
Female Urine	9	6	7	12	31
Male Urine	11	12	2	7	27
NS Urine	0	0	3	0	3
**Total Urine**	**20**	**18**	**12**	**19**	**61**
**Total**	**37**	**33**	**27**	**58**	**142**

NS indicates not specified.

Negative indicates negative for CT, NG, and TV on the Hologic Panther Aptima.

a Columns do not add up to the Total Samples Evaluated because some samples tested positive for more than one pathogen.

**Table 2:** Test performance of the Abbott Alinity m STI assay for the detection of *Chlamydia trachomatis*, *Neisseria gonorrhoeae*, and *Trichomonas vaginalis* by organism against the comparator, Hologic Panther Aptima assay.

	Patient Infection Status Hologic Panther Aptima			Positive Percent Agreement (%) (95% Confidence Interval)	Negative Percent Agreement (%) (95% Confidence Interval)
	Positive	Negative	Total		
***C. trachomatis***				100.0 (90.6–100.0)	99.1 (94.8–100.0)
Positive	37	1	38		
Negative	0	104	104		
Total	37	105	142		
***N. gonorrhoeae***				100.0 (89.6–100.0)	99.1 (94.9–100.0)
Positive	33	1	34		
Negative	0	107	107		
Total	33	108	141*		
***T. vaginalis***				96.3 (81.7–99.8)	99.1 (95.2–100.0)
Positive	26	1	27		
Negative	1	112	113		
Total	27	113	140**		

* Sample total does not equal 142 because 1* and 2** samples were excluded from positive tallies based on presumptive sample degradation, initially positive for the specific pathogen on Panther Aptima, negative when tested on Alinity, and then negative on retesting on Panther Aptima.

Given the multiplex capacity of the Alinity m STI assay, it was possible to detect coinfections with CT, NG, TV, and/or MG within a single test. Specifically, in the sample cohort, 25 of the 142 samples (17.6%) tested positive for more than one microbe. Nine samples were CT and NG double positive, three CT and TV double positive, and three NG and TV double positive. MG testing, which was not performed on the Aptima comparator based on our current clinical practice, was detected in 19 of the 142 samples (13.4%) using the Alinity assay. More specifically, MG was detected in 24.3%, 25%, and 11.5% of samples positive for CT, NG, and TV, respectively, and 8.6% of samples negative for all three of these pathogens (Table 3). Four samples tested positive for three microbes: two samples (1.4%) for CT, NG, and MG; one sample (0.7%) for CT, TV, and MG; and one sample (0.7%) for NG, TV, and MG.

**Table 3:** Rate of *Mycoplasma genitalium* detection in samples positive for *Chlamydia trachomatis*, *Neisseria gonorrhoeae*, or *Trichomonas vaginalis*, or negative for all three of these pathogens using the Alinity m STI assay.

	N	*M. genitalium* positive n (%)
***C. trachomatis***	37	9 (24.3)
***N. gonorrhoeae***	32	8 (25.0)
***T. vaginalis***	26	3 (11.5)
**Negative for other pathogens**	58	5 (8.6)
**Total**	142	19 (13.4)^a^

a Note that two samples were *C*. *trachomatis* and *N*. *gonorrhoeae* double positive, one sample was *C*. *trachomatis* and *T*. *vaginalis* double positive, and one sample was *N*. *gonorrhoeae* and *T*. *vaginalis* double positive, hence the total is not the sum of the other rows.

The limits of detection (95% detection threshold) for the Alinity m STI assay were determined by serial dilution of standards in pooled urine and swab sample matrix negative for all four pathogens, and were, respectively: CT, ≤5 and ≤ 1; NG, ≤5 and ≤ 5; TV, ≤0.5 and ≤ 0.5; and MG, ≤500 and ≤ 250 genome copies/mL (Table 4).

**Table 4:** Limit of Detection (95% detection threshold) for *Chlamydia trachomatis*, *Neisseria gonorrhoeae*, *Trichomonas vaginalis*, and *Mycoplasma genitalium* in urine and swab matrix.

	Limit of Detection (genome copies/mL)	
	Urine	Swab matrix
***C. trachomatis***	≤ 5	≤ 1
***N. gonorrhoeae***	≤ 5	≤ 5
***T. vaginalis***	≤ 0.5	≤ 0.5
***M. genitalium***	≤ 500	≤ 250

## 4 Discussion

The Abbott Alinity m STI assay appears to have excellent analytical performance and identifies pathogens accurately compared with a well-established comparator. Limits of detection in our study were similar to those stated in the assay product insert (CT 17, NG 7.5, TV 0.1, and MG 165 copies/mL). The calculated positive percent agreement in this study also aligns well with a previously published study that evaluated the percent agreement for CT (98.8%) and NG (98.3%) detected by the Alinity m STI and Abbott m2000 RealTime CT/NG assay in 347 patient samples [14].

The Alinity m STI assay's multiplex capability on a high throughput, flexible instrument offers a compelling new testing modality for sexually transmitted infections. Currently, only CT and NG symptomatic testing and screening of high-risk populations are universally recommended for initial testing. TV and MG testing are generally recommended only for follow-up testing in symptomatic patients [7]. Clinically, the Alinity m assay results could be selectively reported for the pathogens of interest (e.g., CT and NG for general screening purposes). Additional reporting of results for TV and MG could be released depending on clinician orders, either initially or in supplementary requests based on results for CT and NG, and/or lack of adequate response to initial therapy for these pathogens. The quadruplex test thereby simplifies specimen acquisition and workup for symptomatic patients. This capability could be leveraged in turn to forestall disease progression; potential loss of patients to care if testing is performed stepwise; and further transmission [[15], [16], [17]].

This study had several limitations. The specimens used in this study were enriched for positivity for the target analytes based on original Hologic Panther Aptima results and may not be representative of a broader patient population. Patient samples were also received in Hologic Aptima transport medium based on current clinical practice at our institution. Therefore, testing was not performed as a prospective study with specimens collected in the Alinity m multi-Collect Specimen Collection Kit approved for use with the Alinity m STI assay. Nevertheless, performance relative to the Hologic Panther Aptima did not appear to be compromised. The transport media are likely similar lithium lauryl sulfate-based lysis solutions based on chemical components listed in respective material safety data sheets. We also did not use a gold standard comparator but rather a previously FDA-cleared assay used in clinical practice. The samples did not include oropharyngeal or rectal swabs, which are among specimens intended for use with the Alinity m STI assay. We also lacked a comparator assay for MG detection. In addition, the Aptima CT/NG, and TV assays do not have an inhibition control, in contrast to the presence of both internal and cellular controls in the Alinity m multiplex assay. Therefore, the rare observation of Panther negative, Abbott positive samples may also potentially result from unrecognized Panther-specific assay inhibition. More generally, results near the assay cutoff, and, therefore, near the limit of detection of both assays, may by definition show variable results on retesting, potentially accounting for observations in the few samples with discrepancies. Lastly, more samples with multiple infections should be evaluated in the future to clinically validate the Alinity m multiplex detection capacity. Notably, the true burden of MG and its role as a commensal or disease entity are not completely understood, especially as the ability to test for MG in significant cohorts has not been available until recently, and neither TV nor MG are reportable pathogens leading to gaps in our understanding of disease associations and epidemiology [7,8].

## 5 Conclusions

Overall, this study indicates the Alinity m STI assay performs well for detection of CT, NG, and TV compared to a predicate assay and offers an advantage as the first FDA-cleared NAAT to simultaneously test for CT, NG, TV, and MG. Our findings also suggests the assay could help fill gaps in understanding sexually transmitted infection epidemiology such as frequency and importance of MG co-infection, and support future prospective STI studies with well-defined populations and clinical syndromes. The Alinity m STI assay holds promise as a valuable tool to efficiently diagnose STIs and perform STI surveillance.

## Funding

Abbott Molecular provided the Alinity m STI reagents and Microbiologics control material used in our method evaluation study. Abbott had no role in study design, data collection/interpretation, manuscript preparation, or decision to publish.

## CRediT authorship contribution statement

**Colette Matysiak:** Writing – original draft, Writing – review & editing, Formal analysis, Conceptualization, Visualization, Methodology, Investigation. **Annie Cheng:** Conceptualization, Methodology, Investigation, Formal analysis, Writing – review & editing. **James E. Kirby:** Supervision, Conceptualization, Formal analysis, Writing – review & editing.

## Declaration of competing interest

Support was received from 10.13039/100015124Abbott Molecular through a medical education grant unrelated to this study.

## Data Availability

Data will be made available on request.
